# Perceptions of free will in obsessive-compulsive disorder: a quantitative analysis

**DOI:** 10.1186/s12888-018-1985-3

**Published:** 2018-12-27

**Authors:** Lucas J. B. van Oudheusden, Stasja Draisma, Sandra van der Salm, Danielle Cath, Patricia van Oppen, Anton J. L. M. van Balkom, Gerben Meynen

**Affiliations:** 1 Department of Psychiatry, Amsterdam Public Health, Amsterdam UMC, Vrije Universiteit Amsterdam, GGZ inGeest, Oldenaller 1, 1081 HJ Amsterdam, The Netherlands; 2 Brain Center Rudolf Magnus, Department of Neurology and Neurosurgery, University Medical Center Utrecht, Utrecht, The Netherlands; 3 Stichting Epilepsie Instellingen Nederland (SEIN), Zwolle, The Netherlands; 4 Department of Clinical and Health psychology, Altrecht Academic Anxiety Centre, Utrecht University, Utrecht, The Netherlands; 5 Faculty of Humanities, Vrije Universiteit Amsterdam, Amsterdam, The Netherlands; 6 GGZ inGeest, Amsterdam, The Netherlands; 7 Willem Pompe Institute for Criminal Law and Criminology, Utrecht University, Utrecht, The Netherlands

**Keywords:** Obsessive-compulsive disorder, Perceptions of free will, Phenomenology, Insight, Quality of life

## Abstract

**Background:**

The aim of this study was to explore perceptions of free will in the repetitive behaviors of patients with obsessive-compulsive disorder (OCD) and to explore their relation with core clinical characteristics.

**Methods:**

Experiences of free will were assessed with the Symptomatology And Perceived Free will rating scale (SAPF) in 295 subjects with a lifetime diagnosis of OCD. Patients’ scores on the SAPF were subjected to an explorative principal axis factor analysis (PAF). Factor scores were regressed on five OCD symptom dimensions and on seven clinical variables: illness duration, severity of OCD, insight, anxiety and depression, suicidal ideation and quality of life.

**Results:**

The PAF revealed three factors: the perceived ability to control and change one’s course of action when faced with an obsession or compulsion (the “alternative possibilities” factor); the experience of obsessions or compulsions as intentional (the “intentionality” factor); and the experience of being the source or owner of the obsessions or compulsions (the “ownership” factor). Lower scores on the “alternative possibilities” factor were associated with lower scores on the washing dimension (β = 0.237, *p* = 0.004) and higher scores on the precision dimension (β = − 0.190, *p* = 0.025) and independently associated with longer illness duration (β = − 0.134, *p* = 0.039), higher illness severity (β = − 0.298, *p* < 0.001) and lower quality of life (β = 0.172, *p* = 0.046). Lower scores on the “intentionality” factor were independently associated with lower quality of life (β = 0.233, *p* = 0.027). Higher scores on the “ownership” factor were associated with higher scores on the precision dimension (β = 0.207, *p* = 0.023) and independently associated with poorer insight (β = 0.170, *p* = 0.045).

**Conclusions:**

The most notable finding of this study is that a diminished experience of free will in OCD is associated with core clinical characteristics: illness duration and severity, insight and quality of life.

## Background

Obsessive-compulsive disorder (OCD) is a psychiatric disorder that is characterized by the presence of obsessions and/or compulsions. In the Diagnostic and Statistical Manual of Mental Disorders (DSM), obsessions are defined as recurrent and persistent thoughts, urges, or images that are experienced as intrusive and unwanted; compulsions are defined as repetitive behaviors or mental acts that an individual feels driven to perform in response to an obsession or according to rules that must be applied rigidly [1]. The definitional terms “intrusive”, “unwanted”, “driven” and “must” suggest that the notion of free will plays a central role in the phenomenology of OCD. More specifically, they suggest that, in OCD, the experience of free will and the capacity to act freely are somehow affected. Indeed, OCD patients are often painfully aware of the sharp contrast between the unexpected and unwanted intrusion of obsessional thoughts or urges and the performance of compulsive rituals on the one hand, and their normal experience of having “free” thoughts or urges and performing “free” acts on the other hand. The experience of a significant loss of freedom and control over one’s thoughts and actions lies at the heart of the suffering of OCD patients [2].

The concept of free will has been studied and debated extensively by philosophers for the past two millennia [3]. Although these studies have given rise to many different perspectives, Walter [4] discerns three main aspects of free will in the contemporary philosophical debate. According to his interpretation, acting out of free will can be conceived of in the following ways: 1. having the ability to act otherwise, that is, having alternative possibilities open to one; 2. acting or choosing for (understandable) reasons*,* which implies intentional behavior; 3. being the owner or (causal) source of one’s actions [5].

When discussing conditions of diminished freedom, philosophers often invoke mental disorders – and OCD specifically – as examples. Regarding OCD, Levy [6] writes:

*We understand that a person suffering from obsessive-compulsive disorder, spending all day washing his hands and checking dozens of times that he remembered to lock the front door, cannot be thought of as having free will. His actions are mechanically dictated by stereotyped scripts, from which he cannot escape. Thus, obsessive-compulsive disorder is a malady of free will.*

Given the importance of the experience of (diminished) free will in OCD phenomenology, it is surprising that the topic has received little attention in empirical research. The present study is designed to address this gap in the literature. Thus, our main aim is to shed new light on what it means to be obsessed and to be compelled in the context of OCD, by exploring perceptions of free will in patients with OCD using insights derived from the philosophy of free will. Having a clearer view on these perceptions might encourage professionals to explore the experiential realm of their patients with them, in addition to the more standard diagnostic approach of assessing symptoms, illness severity, symptom subtypes and comorbidity. The second aim of our study is to explore the clinical significance of free will experience in OCD. Because OCD is a heterogeneous disorder with respect to its thematic content, we first investigated the association between different OCD symptom dimensions and experiences of free will. Furthermore, we assessed the association between experiences of free will and seven clinical variables that were selected based on the a priori hypothesis that they might influence, or be influenced by, experiences of free will: illness duration, OCD severity, insight, severity of comorbid anxiety and depression, severity of suicidal ideation and quality of life.

## Methods

### Design and setting

The present study is embedded within the Netherlands Obsessive Compulsive Disorder Association (NOCDA) study, a multicenter naturalistic cohort study designed to investigate the long-term course and outcome in OCD. The study design and baseline characteristics of the study sample are described in detail elsewhere [7]. In short, at baseline, we included 419 patients with a lifetime diagnosis of OCD, as determined by the administration of the Structured Clinical Interview for DSM-IV Axis I Disorders (SCID-I) [8]. Baseline measurements took place between 2005 and 2009 and included validated semi-structured interviews and self-report questionnaires to gather information on a broad range of variables related to (amongst others) OCD, comorbidity and psychosocial consequences. All included participants were contacted at 2-, 4- and 6-year follow-up, irrespective of their treatment status. The rating scale used to assess free will experience (see below) was administered during the 4-year follow-up wave, in which 295 subjects participated. The study was approved by the Medical Ethical committee of the VU Medical Centre, Amsterdam.

### Questionnaire

**Fig. 1:**
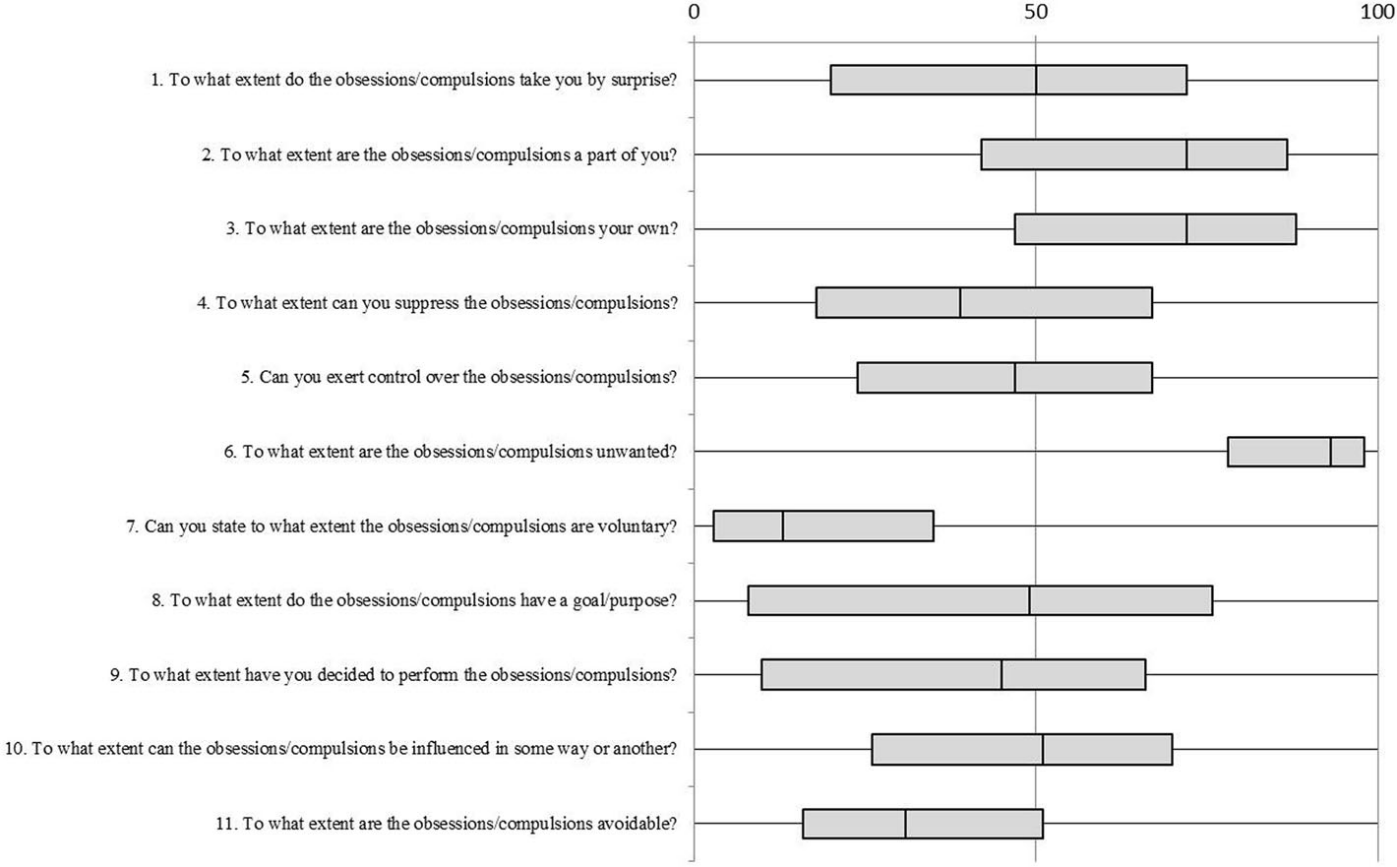
Answers to the 11 questions of the SAPF for the whole study sample. The bars are boxplots, with the central line representing the median score, the edges of the boxes representing the first and third quartile, respectively, and the whiskers extending to the minimum and maximum values. A score of “0” means “not at all”, and a score of “100” means “completely”. Note that for most questions a score of “0” implies minimal freedom, whereas for questions 1 and 6, a score of “0” implies maximal freedom

The Symptomatology And Perceived Free will rating scale (SAPF) is a self-report questionnaire that operationalizes and applies insights from the philosophy of free will as captured in the conceptual framework by Walter [see introduction] to the study of psychopathology. It was originally developed by Van der Salm et al. to study perceptions of free will in a range of movement disorders, including tic disorder [9]. The fifteen items that make up the questionnaire all capture different aspects of free will experience. Because tic disorder and OCD share several important features with regard to phenomenology, comorbidity, underlying neurobiology and treatment strategies [10, 11], the SAPF was considered to be a suitable starting point for the exploration of free will perceptions with regard to obsessive-compulsive behavior. For this study, the wording of the fifteen items was adapted to enable specific application to obsessions and compulsions, without altering any further content. The questionnaire starts by asking the patient whether he or she suffers the most from obsessions or from compulsions. The patient is then requested to fill in the questionnaire with his or her most prominent symptom in mind. Answers to all but one of the questions are given on a Visual Analogue Scale, ranging from 0 (“not at all”) to 100 (“completely”). For the present analysis, we excluded one question that was dichotomous in nature and three questions that dealt with general perceptions of free will and not with symptom-specific experiences, leaving 11 items. See Fig. 1 for the individual items.

### Clinical characteristics

The Padua Inventory-Revised (PI-R) [12, 13] was used to assess the presence and severity of five OCD symptom dimensions: (I) impulses, (II) washing, (III) checking, (IV) rumination and (V) precision. Illness duration was defined as the interval between the age at interview and the reported age at onset of OCD as assessed with the SCID-1 [8]. Severity of obsessive-compulsive symptoms was measured with the Yale-Brown Obsessive-Compulsive Severity Scale (Y-BOCS; Range 0–40; Cronbach’s α for this sample: 0.926) [14]. The degree of insight into OCD symptoms was assessed with the Overvalued Ideas Scale (OVIS; Range 0–10; Cronbach’s α: 0.663) [15]. The Beck Anxiety Inventory (BAI; Range: 0–63; Cronbach’s α: 0.933) and Beck Depression Inventory (BDI; Range: 0–63; Cronbach’s α: 0.912) were used to assess severity of comorbid anxiety and depressive symptoms respectively [16, 17]. Severity of suicidal ideation was measured with the Beck Scale for Suicidal Ideation (BSS; Range: 0–38; Cronbach’s α: 0.872) [18]. Quality of life was assessed with the EQ-5D, yielding a utility score ranging from − 0.59 to 1.00 [19].

### Statistical analyses

Scores on the 11 items of the SAPF for the whole study sample were explored with descriptive statistics. Differences in the mean scores on the 11 items between the group of patients who suffered most from obsessions and the group of patients who suffered most from compulsions were assessed with independent-samples t-tests.

To investigate the interrelatedness of the 11 items of the SAPF, the individual item scores were statistically modelled as the expression of one or more latent factors. To extract the factor structure, an exploratory principal axis factor analysis (PAF) was performed. A preliminary analysis revealed that the Kaiser-Meyer-Olkin (KMO) measure of sampling adequacy was acceptable (0.722). Bartlett’s test was significant and the determinant of the correlation matrix was 0.062, indicating that the matrix was suitable for PAF. To facilitate interpretation, the scores on item 1 and item 6 were recoded, so that a score of “0” signifies “no freedom/control” and a score of “100” signifies “maximum freedom/control”, similar to the other nine items. Missing data were dealt with through listwise deletion: 35 subjects had missings on all SAPF items, and an additional nine subjects had missings on one or more (but not all) SAPF items, leaving a final study sample of 251 subjects. The decision regarding the number of factors to retain was based on the factor eigenvalues (using Kaiser’s criterion of > 1) and the scree plot. To facilitate interpretation, oblique rotation (direct oblimin) was applied. Given the sample size, factor loadings of 0.32 and larger were considered to be significant [20]. Items were allocated to a factor based on their highest loading. The internal consistency of the factors was assessed with Cronbach’s alpha. Subject-specific factor scores for each of the factors were calculated using the regression method.

The association between experiences of free will and OCD symptom dimensions was assessed by regressing the factor scores for the different PAF-derived factors on the five PI-R dimensional scores, using simple regression analyses, each time correcting for the PI-R total score. To investigate how experiences of free will are related to other important clinical aspects in OCD, the PAF-derived factor scores were regressed on the seven clinical characteristics mentioned above, with multiple regression analyses. In the multiple regression analyses, a forced entry method was used for entering the predictors into the model. *P*-values ≤0.05 were considered to indicate statistical significance. All analyses were performed with SPSS version 22.

## Results

### Sample characteristics

The mean age of the 295 participants was 38.4 years (SD 15.6). 53.9% of the participants were female. The mean age at onset was 18.5 years (SD 9.8), the mean illness duration was 20.5 years (SD 15.7), and the mean current illness severity as measured with the Y-BOCS was 15.4 (SD 9.2), reflecting a moderate illness severity. The mean score on the OVIS was 4.4 (SD 1.3), reflecting an average level of fair insight into the obsessive-compulsive symptoms. The mean score on the BAI was 13.6 (SD 10.9), reflecting mild to moderate levels of comorbid anxiety. The mean score on the BDI was 11.6 (SD 9.8), reflecting low levels of comorbid depression. The level of suicidal ideation, as assessed with the BSS, was minimal (mean score: 1.7 (SD 5.1)). One hundred and thirty subjects (44.1%) reported that their most prominent symptom was an obsession, whereas 67 subjects (22.7%) reported that their most prominent symptom was a compulsion. The remaining 98 subjects (33.2%) did not report a most prominent symptom.

### Perceptions of free will in OCD patients according to the SAPF

The answers to the 11 questions for the whole sample are presented in Fig. 1. Here we highlight several findings. The answers for each of the items ranged from “0” to “100”, indicating that for each question, very diverse perceptions exist within the study sample. Compared to all other items, subjects appeared to be most outspoken and in agreement about the items that assess whether the obsessions or compulsions are unwanted (item 6) and voluntary (item 7). The relatively high median of item 6 and the relatively low median of item 7 and the relatively small distribution of both items indicate that subjects generally experienced their symptoms as highly unwanted and involuntary. The majority of the subjects had scores between 50 and 100 on items 2 and 3, indicating that they tended to experience the obsessions or compulsions as a part of themselves (item 2) and as their own (item 3).

A comparison of subjects whose most prominent symptom was an obsession with those subjects whose most prominent symptom was a compulsion revealed several significant, albeit small, differences. Subjects with predominant obsessions experienced their symptoms as more of a surprise than subjects with compulsions (item 1) (mean obs.: 52.7 vs. mean comp. 40.1, *t* = 3.00, *p* = 0.003). Subjects with predominant compulsions experienced their symptoms more as the result of a decision (item 9) (mean obs.: 34.2 vs. mean comp. 48.4, *t* = − 3.10, *p* = 0.002) and at the same time as more difficult to suppress (item 4) (mean obs.: 44.4 vs. mean comp. 34.7, *t* = 2.50, *p* = 0.012) than subjects with predominant obsessions. There were no significant differences in scores on the other eight items between the two groups.

### Factor structure of the SAPF

Based on the eigenvalues and the scree plot resulting from the PAF, three factors were retained for subsequent analysis (eigenvalues were 3.07, 2.04 and 1.13, respectively). Factor 1 explained 27.9% of the variance, factor 2 explained 18.5%, and factor 3 explained 10.3%. The item loadings per factor are presented in Table 1. Factor 1 is characterized by a high loading of the items concerning the ability to suppress, control, influence, and avoid the obsessions or compulsions (items 4, 5, 10 and 11). These items all focus on the perceived ability of the patient to control and change his or her course of action when faced with an obsession or compulsion. In other words, the items reflect the perceived presence of behavioral alternatives to giving in to the obsessions or compulsions. It therefore seems appropriate to label this factor as the “alternative possibilities” factor. Factor 2 is characterized by a high loading of the items that assess whether the obsessions or compulsions are (in)voluntary, (un)wanted, (non-)purposeful and the result of a decision (items 6, 7, 8 and 9). These items deal with the intentional aspects of obsessive-compulsive behavior, and the factor can therefore be best labeled as the “intentionality” factor. Factor 3 is characterized by a high loading of the items that assess the extent to which the obsessions or compulsions are owned by the person and part of one’s self (items 2 and 3). These items deal with matters of ownership and it therefore seems appropriate to label this factor as the “ownership” factor. The internal consistency and reliability, as measured with Cronbach’s alpha, were good for factor 1 (0.809), limited for factor 2 (0.590) and acceptable for factor 3 (0.692). A closer look at the correlations between the items that constitute factor 2 showed that the cohesion within this factor was mainly driven by the correlation between item 6 (“unwanted”) and item 7 (“voluntary”). Correlations between the three factors were positive for factors 1 and 2 and factors 2 and 3, but negative for factors 1 and 3 (corr (1, 2): 0.151; corr (1, 3): − 0.415; corr (2, 3): 0.115).

**Table 1:** Principal axis factor analysis on 11 SAPF items with item loadings per factor

	Factor 1 *“Alternative possibilities”*	Factor 2 *“Intentionality”*	Factor 3 *“Ownership”*
	Expl. variance: 27.9% Cronbach’s α: 0.809	Expl. variance: 18.5% Cronbach’s α: 0.590	Expl. variance: 10.3% Cronbach’s α: 0.692
4. To what extent can you suppress the obsessions/compulsions?	**0.998**	−0.191	0.166
5. Can you exert control over the obsessions/compulsions?	**0.735**	0.032	0.001
10. To what extent can the obsessions/compulsions be influenced in some way or another?	**0.616**	0.077	−0.081
11. To what extent are the obsessions/compulsions avoidable?	**0.448**	0.094	−0.208
1. To what extent do the obsessions/compulsions take you by surprise?^a^	0.224	0.135	− 0.122
7. Can you state to what extent the obsessions/compulsions are voluntary?	0.217	**0.702**	0.011
6. To what extent are the obsessions/compulsions unwanted?^a^	0.163	**0.609**	− 0.007
8. To what extent do the obsessions/compulsions have a goal/purpose?	−0.179	**0.440**	0.073
9. To what extent have you decided to perform the obsessions/compulsions?	−0.054	**0.436**	0.040
3. To what extent are the obsessions/compulsions your own?	0.060	0.040	**0.785**
2. To what extent are the obsessions/compulsions a part of you?	−0.024	0.101	**0.630**

^a^ *recoded*

bold: given the sample size, factor loadings of 0.32 and larger were considered to be significant [20]

### Association with clinical characteristics

A higher score on the washing subscale of the PI-R was associated with a higher score on the “alternative possibilities” factor (β = 0.237, *p* = 0.004). Conversely, a higher score on the precision subscale of the PI-R was associated with lower score on the “alternative possibilities” factor (β = − 0.190, *p* = 0.025) and a higher score on the “ownership” factor (β = 0.207, p = 0.023). The other OCD symptom dimensions were not associated with any of the SAPF-derived factors.

Associations between the three factors of the SAPF and the seven other clinical characteristics are presented in Table 2. Lower scores on the “alternative possibilities” factor were independently associated with longer illness duration (β = − 0.134, *p* = 0.039), higher illness severity (β = − 0.298, *p* < 0.001) and lower quality of life (β = 0.172, *p* = 0.025). Lower scores on the “intentionality factor” were independently associated with lower quality of life (β = 0.233, *p* = 0.027). Higher scores on the ownership factor were independently associated with poorer insight (β = 0.170, *p* = 0.045).

**Table 2:** Multiple regression of the three aspects of free will experience on clinical characteristics

	Factor 1 *“Alternative possibilities”*		Factor 2 *“Intentionality”*		Factor 3 *“Ownership”*	
	Beta^a^	p	Beta	p	Beta	p
Illness duration	−0.134	0.039*	0.045	0.563	0.130	0.085
Y-BOCS severity	−0.298	0.000*	0.013	0.893	0.015	0.873
Beck Anxiety Index	−0.039	0.663	0.028	0.793	0.024	0.813
Beck Depression Inventory	−0.087	0.418	0.024	0.854	0.155	0.216
Beck Suicide Scale	−0.023	0.772	0.010	0.915	−0.001	0.994
EQ-5D	0.172	0.046*	0.233	0.027*	− 0.015	0.882
OVIS	− 0.076	0.297	0.042	0.630	0.170	0.045*

*Abbreviations: Y-BOCS: Yale-Brown Obsessive Compulsive Scale; EQ-5D: EuroQol – 5 Dimensions; OVIS: Overvalued Ideas Scale*

^a^ *Beta values represent standardized coefficients*

*: significant at p ≤ 0.05

## Discussion

To our knowledge, this is the first study to investigate perceptions of free will in patients with OCD using a questionnaire that allows for quantitative analysis. Our results suggest that the SAPF discerns three aspects of free will experience in OCD: 1. the perceived ability to change one’s course of action when faced with an obsession or compulsion (the “alternative possibilities” factor); 2. the degree to which patients experience that their obsessions and compulsions occur for a reason or as the result of deliberate intent (the “intentionality” factor) 3. the degree to which patients experience that they are the owner or originator of their obsessions or compulsions (the “ownership” factor). The three-factor structure of the SAPF as revealed by the exploratory factor analysis maps fairly well onto Walter’s theoretical framework described in the introduction of this article. The distinct nature of these three aspects is underscored by the fact that they differ in their associations with core clinical aspects of the disorder (see below).

The first aspect of free will experience in OCD is the perceived ability to control and change one’s course of action when faced with an obsession or compulsion. The scores on the SAPF items that load on the “alternative possibilities” factor (items 4, 5, 10 and 11: see Fig. 1) suggest that as a group, OCD patients experience very little freedom to pursue a different course of action when faced with their symptoms. In a recent review on psychosurgery and free will, De Ridder et al. [21] identify behavioral inflexibility as the core problem in OCD and suggest that successful psychosurgery for OCD can “*increase the behavioral options, thereby increasing flexible decision making and free will.*” The importance of behavioral inflexibility in OCD is also reflected in the standard psychotherapeutic approach to OCD; the primary goal of exposure and response prevention is to increase the amount of behavioral options the patient can choose from when he or she is faced with obsessional anxiety [22].

An interesting finding is that the perceived ability to control and change one’s course of action when faced with an obsession or compulsion diminishes as illness duration increases. This is in line with recent studies that suggest that illness development in OCD is associated with a shift away from consciously controlled behavior towards habit formation [for a comprehensive overview, see [23]]. Our results also suggest that patients who experience their obsessive thinking or compulsive acting as the *only* behavioral option instead of one of several behavioral alternatives, tend to have a higher severity of OCD and a lower quality of life (see Table 2). It is likely that the association with OCD severity is at least in part the result of conceptual overlap between the SAPF items that load on factor 1 and several items of the Y-BOCS. The association with quality of life is of interest in light of previous studies that have shown that OCD has a significant impact on the quality of life of patients and that this impact is mainly driven by illness severity and presence of comorbidity [24]. The fact that patients with a diminished experience of having alternative possibilities had a lower quality of life, regardless of their illness severity and comorbidity burden, suggests that free will experience plays an independent role in the way OCD patients perceive their well-being.

The second aspect of free will experience in OCD is the degree to which patients experience that their obsessions and compulsions occur for a reason or as the result of deliberate intent. The group scores on the related SAPF items show that although patients generally perceive their obsessions and compulsions as highly unwanted (item 6) and involuntary (item 7), there is large within-group variation in the extent to which they perceive their behavior as goal-directed (item 8) and as the result of a decision (item 9). A part of the variation in item 9 could be explained by the focus of the questionnaire: compulsions were experienced more as the result of a decision than obsessions. Interestingly, a perceived lack of intent or reason underlying the obsessive-compulsive symptoms was associated with a lower quality of life. The association between OCD and a diminished quality of life has been firmly established in previous research [25]. A consistent finding is that the presence of comorbid depressive symptoms plays an important mediating role [24]. There is also evidence that the presence of compulsions has a stronger negative effect on quality of life than the presence of obsessions [26]. Of note, the association between a perceived lack of intent or reason underlying the obsessive-compulsive symptoms and a lower quality of life was adjusted for the severity of comorbid depression and general illness severity. A tentative interpretation of this association could be that a prolonged experience of being forced to perform empty, pointless rituals has a negative impact on one’s sense of purpose and meaning in life. However, it should also be noted that the limited psychometric quality of factor 2 (see Results section) puts the validity of this association into question.

The third aspect of free will experience in OCD is the degree to which patients experience that they are the owner of their obsessions or compulsions. The group scores on the related SAPF items show that patients exhibit a strong sense of ownership or belonging with regard to their symptoms (items 2 and 3: see Fig. 1). The experience of self-ownership is indeed recognized as a core phenomenological feature of OCD [27]. As a sense of ownership can be seen as a prerequisite for mastery over one’s actions (and therefore also one’s symptoms), we have so far considered it as an indicator of free will. In this context, the finding that patients with poorer insight tend to have a heightened experience of being the owner of their symptoms is of interest. It might be that this factor of the SAPF actually measures the degree of egosyntonicity, that is, the degree to which a patient has incorporated his or her symptoms into his or her self-image or identity. Clinical experience suggests that rather than *increasing* free will, egosyntonicity actually makes it harder for patients to distance themselves from their symptoms and thereby can lead to a *decrease* in mastery and free will. The fact that a *heightened* experience of being the source of the symptoms is associated with a *diminished* experience of having alternative possibilities also questions the assertion that a sense of ownership indicates free will in the context of OCD. The finding that a higher score on the precision subscale of the PI-R was associated with a heightened experience of being the source of the obsessions or compulsions fits with the empirical finding that patients with prominent precision symptoms often have a comorbid obsessive-compulsive personality disorder [11] and experience their symptoms as egosyntonic.

The conceptual distinction presented in this study might have several clinical implications.

First of all, a more nuanced understanding of what it means to be obsessed and compelled in the context of OCD may help healthcare professionals to better empathize with the challenges that OCD patients face. This could improve the quality of the therapeutic relationship, as many patients with OCD experience significant shame and confusion with regard to their own symptoms. Secondly, the incorporation of these concepts in the clinical discourse might enrich the practice of cognitive behavioral therapy. Exploring experiences that are at the core of the patient’s distress might help them to let go of long held dysfunctional beliefs and increase motivation to engage in behavioral experiments. Finally, the inclusion of these concepts in follow-up consultations might expand the ways in which professionals and patients track changes during treatment, above and beyond measuring scores on an illness severity rating scale.

Strengths of this study include the large sample size and the detailed characterization of the cohort. Several limitations have to be addressed. The fact that this is the first study to apply the SAPF to a population of OCD patients warrants caution when interpreting the results. Future studies are needed to further assess the validity of the questionnaire for this specific population. In addition, the reliability analysis shows that the psychometric quality of the SAPF could benefit from adjustments to the items that constitute the “intentionality factor” (factor 2).

In our opinion, two directions for future research seem particularly promising. First, a more detailed analysis of differences in perceptions of free will between obsessions and compulsions would be very informative. In the current design of the study, subjects are asked to rate free will perceptions for either their main obsession or their main compulsion, which limits the comparison of free will perceptions regarding obsessions versus compulsions to between-person differences. An interesting future modification to the design of the SAPF might be to ask subjects to rate free will perceptions with regard to both their main obsession and their main compulsive behavior. This would result in within-person differences in free will perceptions, that can then be compared across the entire sample. Such a comparison could shed new light on the longstanding debate about the functional nature of compulsive behavior. Traditionally, compulsive behavior is distinguished from other types of stereotyped or repetitive behaviors by referring to the functional nature of compulsive behavior (in contrast to the random nature of other repetitive behaviors). Compulsive behavior is seen as a goal-directed effort on the part of the patient to reduce the anxiety or distress that is evoked by obsessions [28]. It is for this reason that in previous editions of the DSM, OCD was classified as an anxiety disorder. Current perspectives on compulsive behavior tend to de-emphasize its anxiety-driven character [29, 30], which is reflected in the decision to remove OCD from the group of anxiety disorders in the latest edition of the DSM [1]. By distinguishing free will perceptions with regard to obsessions and compulsions within the same subject, one could test the hypothesis (along more traditional lines) that patients who report more obsessional anxiety also have a stronger experience of reason or intent underlying their compulsive behavior. Furthermore, as the washing and checking symptom dimension is thought to be more anxiety-driven than other OCD symptom dimensions [31], one could test the hypothesis that subjects who score high on these dimensions also have a strong experience of intent underlying their compulsive behavior.

A second interesting direction for future research would be to track within-person changes in free will perceptions over time. Repeated assessments in a naturalistic context could shed more light on the effect of prolonged illness duration on perceptions of intent and ownership accompanying compulsive behavior. Repeated assessments in a treatment context could shed light on the role these (changing) free will perceptions play in the improvement of quality of life that usually accompanies successful treatment.

## Conclusions

To conclude, the present study sheds new light on what it means to be obsessed and to be compelled in the context of OCD by distinguishing three aspects in the experience of free will in this disorder; namely, the perceived ability to change one’s course of action when faced with an obsession or compulsion; the experience of obsessions or compulsions as intentional; and the experience of being the source or origin of the obsessions or compulsions. The most notable finding of this study is that a diminished experience of free will in OCD is associated with important clinical parameters; in particular illness duration and severity, insight and quality of life. The clinical relevance of the conceptual distinction presented in this study lies in the positive impact it might have on the therapeutic relationship between professional and patient and the enrichment it might offer to the practice of cognitive behavioral therapy and the process of tracking changes during treatment. Future studies could focus in more detail on differences in free will perceptions between obsessions and compulsive behaviors, and longitudinal assessment of changes in free will perceptions during treatment. The results of the present study suggest that the experience of free will in OCD is a clinically relevant avenue for future investigations.

## Abbreviations

BAI: Beck anxiety inventory
BDI: Beck depression inventory
BSS: Beck scale for suicidal ideation
DSM: Diagnostic and statistical manual of mental disorders
EQ-5D: EuroQol – 5 dimensions
KMO: Kaiser-meyer-olkin measure of sampling adequacy
NOCDA: Netherlands obsessive compulsive disorder association
OCD: Obsessive-compulsive disorder
OVIS: Overvalued ideas scale
PAF: Principal axis factor analysis
PI-R: Padua inventory-revised
SAPF: Symptomatology and perceived free will rating scale
SCID-1: Structured clinical interview for DSM-IV Axis I disorders
Y-BOCS: Yale-brown obsessive-compulsive severity scale
